# The Fitness Effects of Love

**DOI:** 10.1371/journal.pbio.1002249

**Published:** 2015-09-14

**Authors:** Roland G. Roberts

**Affiliations:** Public Library of Science, Cambridge, United Kingdom

## Abstract

A study of zebra finches reveals the potential advantages of idiosyncratic mate choice in monogamous animal species. Read the Research Article.

Humans are a mostly monogamous species—a large proportion of us choose mates with whom we spend long periods of time, and many of us produce children through sexual reproduction with those mates. We tend to think of our choice of mate as being very specific—the result of a long and judicious screening process involving nervous flirtations, set-ups by friends, online matchmaking sites, awkward dates, humiliating rejections, hasty retreats, and the occasional lucky strike. The eventual relationship, after whittling down a cast of thousands, is founded on love, and can be a thing of wonder.

But we are also biological entities, subject to the cruel pressures of natural selection—isn’t this choosiness rather a costly waste of time and energy when we should be just “going forth and multiplying?” Naively, perhaps if we somehow end up selecting the genetically “best” mates, then choosiness might pay off, but not everyone can end up with the “best,” and much of the time our choices can seem, frankly, rather idiosyncratic (“What on earth does she see in him?”). What, if anything, is the evolutionary point of it all?

Doing a cost/benefit analysis of love is a challenging business, with many potential confounds, and—in the case of humans—some ethical constraints with regard to the possibilities of experimentation. A new study published in *PLOS Biology* by Malika Ihle, Bart Kempenaers, and Wolfgang Forstmeier attempts to use a model animal in an elegant experiment designed to tease apart the reproductive consequences of mate choice.

The authors took advantage of the fact that the zebra finch (*Taeniopygia guttata*, a native bird of Australia; Fig 1) shares many characteristics with humans, mating monogamously for life and sharing the burden of parental care. It was already known that the female finches choose mates in a way that is specific to the individual, and there is little consensus among females as to who is the cutest male.

**Fig 1:**
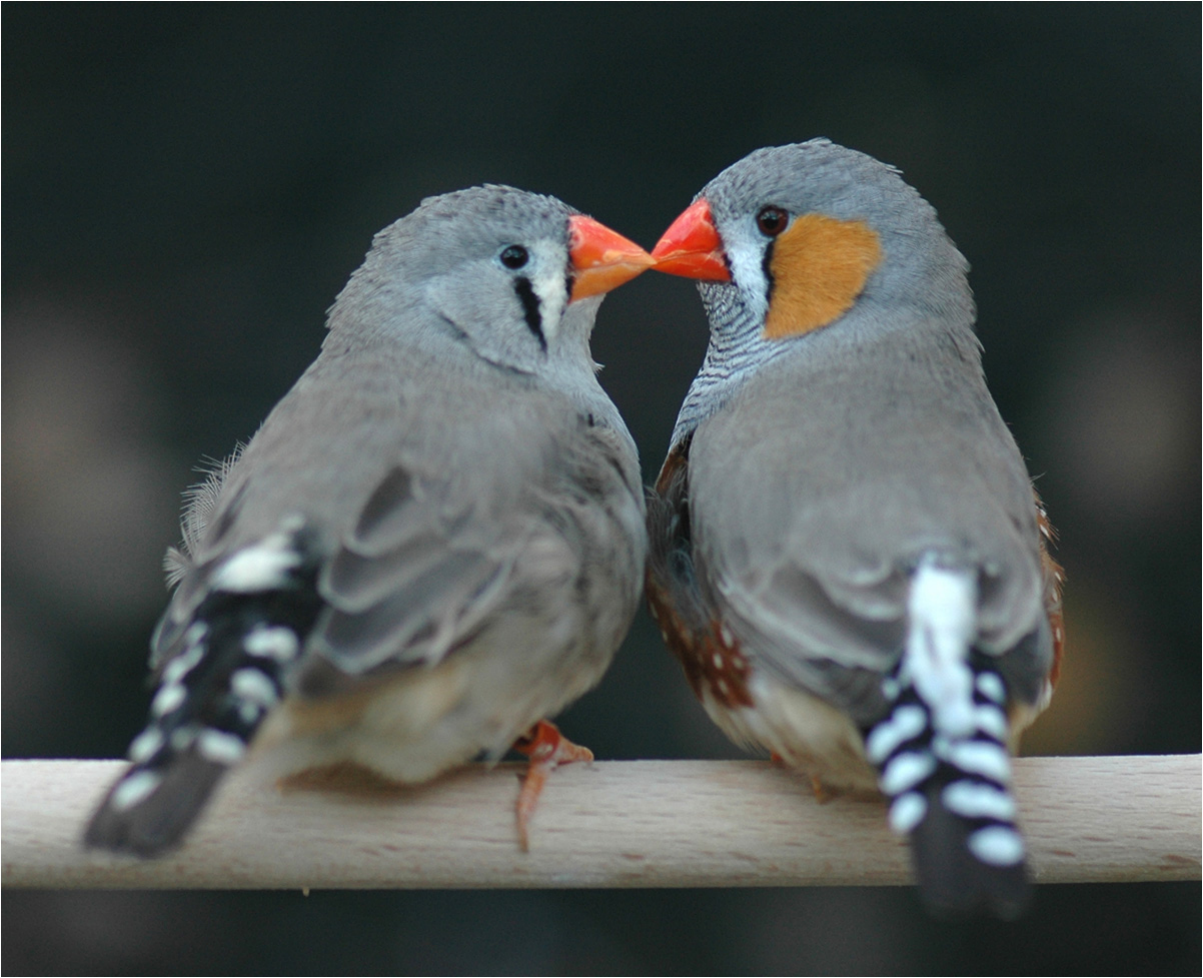
Idiosyncratic love: What do they see in each other? In zebra finches, individuals’ mating preferences appear random and hard to understand but can lead to major fitness consequences. *Image credit*: *Wolfgang Forstmeier*.

Using a population of 160 birds that had recently been isolated from the wild, the authors set up a speed-dating session, leaving groups of 20 females to choose freely between 20 males. Once the birds had paired off, half of the couples (the “chosen” or C group) were allowed to embark on a life of wedded bliss. For the other half, however, the authors intervened like overbearing Victorian parents, splitting up the happy pair and forcibly pairing them with other broken-hearted individuals (the “non-chosen” or NC group).

Bird couples, whether happy or somewhat disgruntled, were then left to breed in aviaries containing six pairs (three chosen, three non-chosen). The authors assessed couples’ behavior and the number and paternity of dead embryos, dead chicks and surviving offspring. In order to rule out certain possible explanations, some couples were subjected to a further round of mate-swapping before the next breeding season.

Strikingly, the overall reproductive fitness (measured as the final number of surviving chicks) was 37% higher for individuals in chosen pairs than those in non-chosen pairs. However, because reproductive fitness is the final read-out from a chain of separate contributing influences, this begged the question of exactly how mate choice was having its effects on fitness.

The females of both groups laid the same number of eggs, suggesting that their initial investment isn’t affected by the mismatched mate. But the nests of non-chosen pairs had almost three times as many unfertilized eggs as the chosen ones, and a greater number of eggs that were neglected (either buried or lost).

Based on previous experiments in which eggs laid by one pair of birds are fostered by another pair, the authors knew that embryo mortality is largely determined by genetic incompatibility between parents, and hatchling mortality by behavioral incompatibility. Comparing these two phenomena between chosen and non-chosen pairs gave a clear answer—embryo mortality was the same in each group, but the mortality of the hatched chicks was markedly higher in the non-chosen couples. This seemed to suggest that behavioral incompatibility between the non-chosen parents—and not genetic incompatibility—might be driving the reduced reproductive fitness.

So what form might this behavioral incompatibility take? Watching the couples’ courtship showed some differences: although non-chosen males paid the same amount of attention to their mates as the chosen ones did, the non-chosen females were far less receptive to their advances, and tended to copulate less often. A multicomponent analysis of harmonious behavior (friendliness, mutual following, synchronous activity) revealed that non-chosen couples were generally significantly less lovey-dovey than the chosen ones.

Looking in greater detail at the increased number of dead chicks in non-chosen pairs, the authors found that most deaths occurred within the chicks’ first 48 hours—the time of maximal attendance from the father—and that non-chosen fathers were markedly less diligent in their nest-care during this critical period.

It should be noted that the birds do go some way to try and undo the researchers’ meddling—there was a higher level of infidelity in birds from non-chosen pairs, and interestingly, the straying of male birds increased as time went by while that of females decreased. Irreversible divorces were more frequent in the assigned marriages than the chosen ones.

The authors’ analysis of their data leads them to the conclusion that the benefits of mate choice are linked to behavioral compatibility. Two principal differences between their chosen and non-chosen groups of courting birds point to how this compatibility might, in turn, be acting to increase reproductive fitness. The first is that non-chosen couples have more infertile eggs; differences in behavioral data suggest that this is because females are less “turned on” by a randomly assigned mate, spurning their advances and declining to copulate. The second is that the non-chosen couples have a poor record of chick survival, and the behavioral data implicate negligent care by the father in the hatchlings’ early days.

Overall, it seems that zebra finches vary rather idiosyncratically in their tastes, and chose mates on the basis that they find them stimulating in some way that isn’t necessarily obvious to an outside observer. This stimulation increases the likelihood of successful copulation and encourages commitment for the time needed to raise young; together these factors maximize the couple’s likelihood of perpetuating their genes. Sounds familiar? This is presumably what the human dating game is about, the need to find a compatible mate perhaps exacerbated by the extended phase of dependence during which our children need parental support. Indeed, these authors’ results are consistent with some studies on the differences between love-based and arranged marriages in human society.
